# Utilization of *Swertia chirayita* Plant Extracts for Management of Diabetes and Associated Disorders: Present Status, Future Prospects and Limitations

**DOI:** 10.1007/s13659-020-00277-7

**Published:** 2020-10-28

**Authors:** Pinaki Dey, Joginder Singh, Jagadish Kumar Suluvoy, Kevin Joseph Dilip, Jayato Nayak

**Affiliations:** 1 Department of Biotechnology, Karunya Institute of Technology and Sciences, Coimbatore, Tamil Nadu 641114 India; 2 Department of Microbiology, Lovely Professional University, Phagwara, Punjab 144411 India; 3 Biotechnology Department, Vignan Foundation for Science Technology and Research, Guntur, Andhra Pradesh 522213 India; 4 National Dong Hwa University, Department of Life Sciences, Hualien, China; 5 Department of Chemical Engineering, VSB Engineering College, Karur, Tamil Nadu 639111 India

**Keywords:** *Swertia chirayita*, Diabetes, Phytochemicals, Conservation

## Abstract

**Abstract:**

Diabetes mellitus is referred as common metabolic abnormalities characterized as hyperglycemia, mainly caused due to insufficient production of insulin at cellular level or/and defects in insulin action. Such an endocrine disorder is responsible for serious health problems and its worldwide prevalence is rapidly increasing. Common management of diabetes by oral administration of drugs without creating any side effects is still considered a challenging task and increasing cost of conventional medicine in developing countries is another matter of concern. To address these issues, traditional preparations of herbal plant extracts in the form of medicines already gained immense attention. *Swertia chirayita* is one among such plants which is known for its hypoglycemic potential. Numerous chemical constituents with promising pharmacological properties have been identified from such plant extracts but still, such compounds have not been well characterized, specifically against human application. Hence, more research efforts are necessitated to understand exact mechanism of such compounds and to develop overall safety of such plant formulations. Present review clearly represents antidiabetic properties of *Swertia chirayita* extract*,* strategies to be taken to strengthen its safety application on humans and biotechnological interventions that ensure conservation of such endangered species to promote its future application in modern medicine.

**Graphic Abstract:**

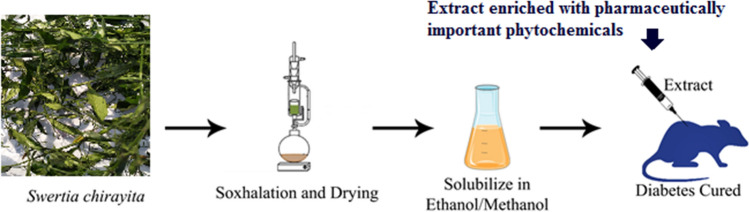

## Introduction

Diabetes mellitus has been considered as most prevalent endocrine metabolic disorders significantly affecting 25% of the world population with morbidity and mortality. It is classified in two different types based on insulin dependency. The total occurrence of diabetes is about 10% of the overall population, and 90% of such disease is considered under type 2 [1]. Diabetes of first category is mostly distinguished by inadequate production or reduced response to key regulatory hormone insulin during body’s metabolism. Insulin is considered as the most essential hormone which helps the conversion of carbohydrates like sugar and starches into energy and lack of production of such hormone leads to increased level of blood glucose. Such a serious endocrine syndrome is characteristically responsible for an increased risk of heart attack, cardiovascular disorders including atherosclerosis, renal failure and other diseases like retinopathy, neuropathy and nephropathy [2]. The diabetic count is expected to reach 300 million or even more at the end of year 2025 according to the prediction of WHO [3]. Common therapies and medicines which are presently available to treat the disease includes insulin and other oral antibiotic agents like sulfonylureas, biguanides, glinides and most of them have several limitations with adverse impacts [4]. Increasing cost of the conventional medicine in developing countries is another crucial factor which enhances the research thrust towards traditional herbal medicine with enhanced hypoglycemic activity. Many indigenous medicinal plants with such therapeutic potential have been found out and their mechanisms of activity have been well studied [5].

Plants have always played a significant role as an ideal resource to generate traditional medicines. About 21000 plants around the world were enlisted by WHO to be used for the medicinal objective and nearly around 800 plants have been specified for antidiabetic potential [6]. Among them, 150 species have received large scientific and medical attention in commercial arena [7]. India, being recognized as botanical garden of the world is the biggest producer of such herbal plants [8]. Presence of the components like glycosides, alkaloids, terpenoids, flavonoids, carotenoids in such medicinal plants are highly effective to trigger function of pancreatic tissues to enhance the production of insulin or to prohibit intestinal adsorption of glucose [9]. Therapeutic potential of different important plant extracts were evaluated through experimental trials using various animal models [4, 10, 11]. Among all such herbal plants, *Swertia chirayita* is very popular and has been well recognized mainly for its anti-hyperglycemic activities. The genus *Swertia* which belongs to family Gentianaceae, has approximately 135 species with herbal remedial properties [12]. Among them, *Swertia chirayita*, popularly known as “*chirayata* or *chirayita*”, is considered to have diverse therapeutic properties, including anti-diabetic, anti-inflammatory, hypoglycemic, hepatoprotective, antibacterial, wound healing, antipyretic, antihelmintic, antioxidant and antitussive [12, 13]. Large number of publications [14–20] in the research field clearly suggests the increasing importance and application of such important medicinal plant in various therapeutic fields. Brahmachari et al. [21] clearly indicated *Swertia* genus as a rich sources of xanthones, flavonoids, irridoid, terpenoids and alkaloids. Bhattacharya et al. [22] specified the presence of twenty precious polyhydroxylated xanthones in such plant extract. Hypoglycemic effects of extracts, developed from various parts of this plant like bark, leaf, shoot, root, and even entire plant were experimentally evaluated by large number of researchers [23–26]. In individual research investigations, Thomson et al. [26] and Kavitha and Dattatri, [25] emphasized the therapeutic potential of aqueous extract of the species whereas Sekar et al. [23] and Alam et al. [22] specifically highlighted the importance of ethanoic extract of the plant for exploitation of its antidiabetic and antioxidant properties. Similarly, anti-carcinogenic, antimicrobial, antihelmintic, antimalarial, anti-hepatitis, analgesic and anti-inflammatory properties of plant extract, formulated from different parts of the plant were experimentally determined by different researchers [20, 27–31].

In spite of its diverse medicinal properties, more specifically as potent antidiabetic herb, this plant did not gain deserving importance in commercial arena. It may be due to deficiency of well-defined specifications or dosages of medicines derived from it, followed by lack of belief on such medicines over commercially known ones. Moreover, insufficient number of studies has been attempted to prove the application of the plant on management of free radicals as well as to define the role of its active ingredients specifically against human diseases. On the other side, increasing usages and demand of the plant species in commercial arena lead to unrestricted over-exploitation, continuous deforestation and unethical overharvesting of the wild plant. Considerable depletion of such valuable bioresources and inadequate efforts for its replacement resulted into new designation of the plant under critically endangered species. The present review aimed to represent the status of major crippling disease, diabetics, detail description of the *Swertia chirayita* in terms of its active ingredients, their properties, effectiveness against diabetics and associated disorders. The review also identifies the strategies to strengthen the applications of the plant extract on human trials and conservation strategies to endorse its future application.

## Diabetics and Its Impact

Diabetes mellitus (DM) is a polygenic disorder, in which body does not deliver enough of the insulin, and/or body develops defects in insulin action which finally causes elevated amount of sugar in bloodstream. The estimated increase in global occurrence of diabetics would be from 4% in 1995 to 5.4% in the year of 2025 [7]. It is estimated that in India, the prevalence of diabetics is high as nearly 33 million adults are affected by the diabetes and at the end of year 2025, the number is expected to reach at 57.2 million [7]. Diabetes is principally characterised by hyperglycaemia which alters lipid, carbohydrates, protein metabolism and it enhances the tendency to get easily affected by serious long-term disorder including cardiovascular diseases [32]. Prolonged hyperglycaemia can lead to severe compilation which probably affects body systems and even patient’s medication [32]. Untreated cases of diabetes shows severe vascular as well as tissue injury which finally comes up with serious health problems like ulceration, neuropathy, retinopathy and cardiovascular compilation [33–36]. Among the two most well-known types of diabetes, Type 1 is dependent on insulin and it is caused due to insufficiency or dysfunction of insulin. The condition is mainly caused by the limitations in activity of beta cells. Whereas, Type 2 diabetics is considered as insulin independent [37] and major treatment procedures are through changes in dietary pattern, workout and medication. Such type of diabetes is comparatively more common as it consists 90% of total diabetic population and usually happens in obese individuals. Both two types of diabetics are associated with hypertension, dyslipidemia, unusual thrust, frequent urination, and extreme hunger with vomiting, weight change, blurred vision, excessive weakness, nausea and mood swings [7]. In addition, diabetes has an indirect relationship with many other endocrine disorders which includes abnormalities in production of proteins and lipids. Extra-cellular proteins such as elastin, collagen, laminin and laminin, which are produced in diabetic patients, are converted to form glycoproteins due to hyperglycemia. Development of complications due to diabetes, like cataracts, microangiopathy, atherosclerosis and nephropathy, normally come up with the alteration of these proteins which exist in associated tissues such as basement membranes, lens and vascular wall. The disease is mainly characterized as hyperglycemia, hypercholesterolemia and hypertriglyceridemia due to insulin secretion defects or reduced tissue sensitivity to insulin. Patients who are unable to make enough insulin are additionally treated with insulin dosages whereas those patients whose cells are unable to respond properly to insulin are treated with different drugs, developed to cure carbohydrate-metabolism disorders. Several therapies have been implemented for the treatment of diabetics but all are having certain restrictions due to their expensive nature and risks for the generation of hypoglycemia, gastrointestinal problems [38]. Considering the recent developments, efforts are being made to develop natural, economic and effective herbal medicine, having suitable antidiabetic property and fewer side effects.

## Medicinal Plants with Anti-diabetic Potential

Medicinal herbs have consistently played a significant role in managing various diseases as they are considered as very good resources of therapeutically valuable phytochemicals. Plants are traditionally used throughout the world for various treatments and many drugs have been isolated from them in the form of different extract. In recent years, many researches indicated the utilization of various medicinal plants to treat diseases in a specific part of the body system, such as the nervous system, cardiovascular systems, respiratory systems, digestive systems as well as in different body organs. Cultural and geographical factors of different plant also facilitates the management of various diseases in different forms, such as in the form of crude extracts, whole plants, plant pastes, infusions, etc. [39]. Nutritionally valuable herbal plants are now used simultaneously for both food and medicinal purposes [40]. Various parts of the herb which have been used for the management of diabetes mainly includes leaves, barks, seeds, fruits, stems, flower and in some cases whole plant itself. According to recent studies, 30000 plant species have been utilized for therapeutic intensions over the world and among them 800 plants specifically poses anti-diabetic properties [2]. A certain number of indigenous herbal plants have been evaluated by using several experimental procedures in order to assess their therapeutic efficacy [4, 5, 7]. Herbal medicines are already employed for the treatment of polygenic diseases in developed nations where the expenditure of standard medicines was not totally affordable for general population. Confirmation of hypoglycemic effect of some medicinal plant extracts has been made against second type of diabetes by using human cell lines and animal models. Presence of the components like polysaccharides, peptidoglycans, hypoglycans, glycosides, guanidine, steroids, carbohydrates, glycopeptides, terpenoids and alkaloids with antidiabetics properties have been confirmed in such type of extracts [5]. Continuous investigation on medicinal properties of such extract is also recommended by expert committee members of WHO. In general, total consideration of herbal medicine in present healthcare system is still difficult due to inadequate presence of scientific and clinical evidences related to their effectiveness and safe application. To overcome such condition, necessity is there to perform more clinical practices on herbal drugs through applying various animal models and increasing awareness among common people is also required about ongoing researches related to the field.

## *Swertia chirayita* and Its Valuable Constituents

*Swertia chirayita* is a popular ancient herbal plant which became familiarized to Europe in 1839. The plant is familiar for its multiple names like Nidrari, Haima, Ramasenka, Kairata (in Sanskrit), Chiravata (in Urdu), Chireta (in Bengali) and Qasabuzzarirah (in Arabic and Farsi) [41]. The plant mainly matures at tall altitudes in sub-temperature regions specifically slopes of Himalayan shady places, typically between the altitudes of 1200–1500 m from Bhutan to Kashmir [12, 42]. The places, where the plant is also cultivated are middle part, Meghalaya and Khasi hills of India. The genus is well known in Chinese traditional medicinal practices and twenty of such species from the same genus were being implemented for treating hepatic, inflammatory and choleric diseases [21]. Total forty endangered species of *Swertia chirayita* have been identified in India and they are mostly annual or biennial herb [43, 44]. Such medicinal herb grows maximum up to 0.5–1.5 m. Leaves of such plant are lanceolate, has a cordate base, containing five to seven nerves, almost oppositely oriented and almost 4 cm length [45]. The root of such medicinal herb is simple, yellowish in colour, somewhat oblique, almost 7–8 cm long whereas flowers look small, numerous, tetramerous and green-yellowish in colour [46].

**Fig. 1:**
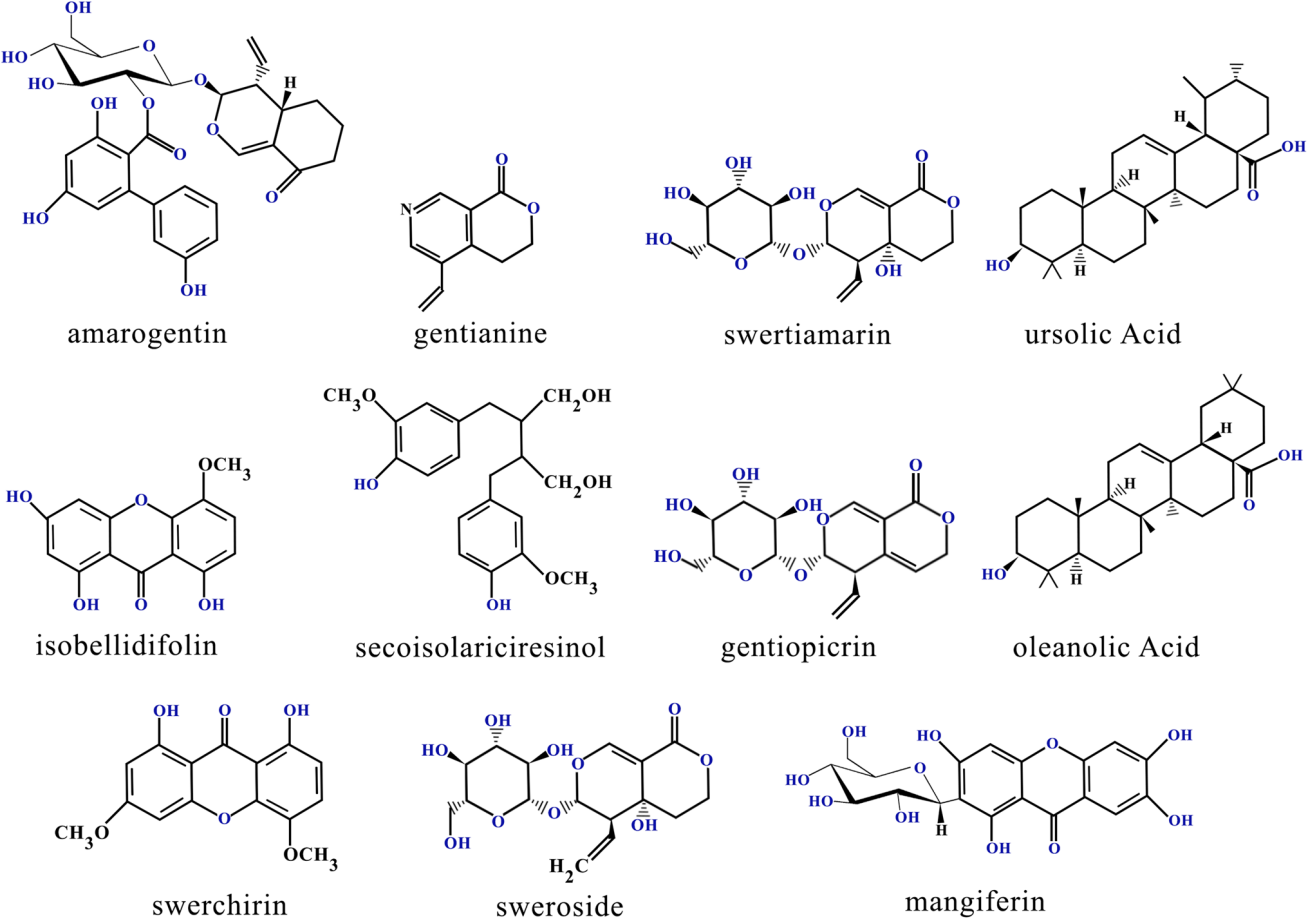
Chemical structures of most important phytochemicals obtained from *Swertia chirayita*

The plant consists of plenty of pharmaceutically important phytochemicals like xanthones, ursolic acid, flavonoids, terpenoids, iridoids, amarogentin, glycosides, secoiridoids and swertiamarin. Ophelic acid (C_13_H_48_O_15_) and chirantin (C_26_H_48_O_15_) are two major compounds present in such plant, are reasonable for developing bitter test. In terms of nature, ophelic acid is yellow in colour, non-crystalline, hygroscopic in characteristics and can be dissolved in water, ether or alcohol. Chirantin or Amarogentin binds with tannic acid to form insoluble compound. The compound is pale-yellow in colour, soluble in alcohol, ether, specifically in warm water. The compound splits into ophelic acid, water and *chiratogenin* (C_13_H_24_O_3_) when it is boiled with hydrochloric acid [47]. It represents chemo-preventive and anti-leishmanial effects and possess properties like topoisomerase inhibition [48]. Presence of transparent monoterpene alkaloid like Gentianine is also proved in *S. chirayita* extract. The compound belongs to the series of compounds which are recognized as pyranopyridines, polycyclic aromatic compounds containing pyran ring attached with a pyridine ring. In terms of nature, gentianine is soluble in aqueous solution and known for its anti-inflammatory, anaesthetic, antihistamine, anticonvulsant properties [49]. It also poses other medicinal properties like antimalarial, antiamoebic, hypotensive, antipsychotic, and antibacterial properties [50]. Properties like bitter taste forms gentianine a potential biomarker for ingesting of such compound containing food product. Swertiamarin which is considered as a secoiridoid glycoside, present in the same plant extract, is known for its analgesic property. The compound was successfully used to treat diseases like arthritis, malaria, diabetes, hypertension, atherosclerosis and abdominal ulcers [51]. Such potential secoiridoid glycoside was successfully reported to control increased serum total cholesterol, triglycerides in high cholesterol induced rats. Swerchirin is one of the member classes of xanthones, isolated from *Swertia* spp., possess similar medicinal properties like hypoglycemic, antimalarial and antihepatotoxic activity. Similarly like Swerchirin, presence of nine tetraoxygenated xanthones were also proved in *Swertia* spp. Preliminary pharmacological screening clearly revealed the presence of such xanthones in the plant extract and it mainly signifies its therapeutic potential. Mangiferin, another potential bioactive constituent present in chirayita species, known for its antioxidant properties and other versatile therapeutic potentials [52]. The compound also possess health endorsing antimicrobial, antiallergic, anticancer, hypocholesterolemic and immunomodulatory properties [53]. The compound was proved to have inhibitory mechanism against various human cancers. Poor solubility, mucosal permeability and bioavailability of the compound mainly hinders its development as most successful clinical therapeutic agent and physicochemical modification is required to further extend its application. Lignan are the another type of fiber-associated polyphenolic substances normally isolated from the same plant origin. The compound is hepatoprotective in nature and basically obtained from a large variety of plant associated food products including seeds, grains, nuts, vegetables, legumes, fruit and drinks such as wine, tea or coffee. Existing researches in this field suggest that food rich in lignans are beneficial for health and cure diseases but additional investigations are required to justify the specific role of lignans. Along with such compounds, *chirayita* also contains verities of triterpenoids including pichierenol, swertanone, gammacer-16-en-3β-ol, 21-a-H-hop-22(29)-en-3β-ol, taraxerol, swertenol, oleanolic acid, ursolic acid, episwertinol, swerta-7, 9(11)-dien-3β-ol [41]. Existence of some specific pentacyclic triterpenoids like β-amyrin, friedlin, chiratenol, kairatenol is also proved in this plant species. Present researches clearly reported the application of such naturally producing triterpenoids against several human health compilations which includes different cancers. Taraxerol as well as oleanolic acid are well known for their analgesic properties whereas ursolic acid was identified for its chemoprotective, anti-inflammatory and anti-microbial characteristics [54]. Different types of experimental animal models have been already implemented to establish anticancer, anti-proliferative and pro-apoptotic properties of different triterpenoids present in the same plant species. Researchers like Basnet et al. [55] evaluated the hypoglycemic potential of bellidifolin, isolated from *Swertia japonica* (another *Swertia* species) on STZ-induced diabetic rats. The compound was highly effective to reduce glucose level in normal and diabetic rats when the compound was orally and intraperitoneally administrated. Previously Ghosal et al. [56] recognized the presence of nine tetraoxygenated xanthones in roots and aerial parts of *Swertia chirata* and among those components, 1,5,8-trihydroxy-3- methoxyxanthone was identified with blood sugar lowering ability. Existence of anti-inflammatory xanthone derivative (1,5-dihydroxy-3,8-dimethoxyxanthone) was also recognized in the same plant species and activity level of the compound was evaluated by using sub-acute and chronic male albino rat experimental models [57]. Chemical structures of most specific and important therapeutically active phytochemicals obtained from S. *chirayita* plant extract are represented in Fig. 1.

## Therapeutic Potential of *Swertia chirayita*

The medicinal plant has been utilized by different indigenous population groups for several clinical applications through multiple ways. People widely used the total plant as household remedy for treatment and control of vomiting, hepatitis, constipation, inflammation, weak stomach and indigestion. Generally the plant has been utilized for remedial purposes of malaria, chronic fever, urine disorders, hypertension, certain type of mental disorders, anemia, skin disease, bronchial asthma, liver disorder, gastritis, worms, specific ulcers, purification of blood and tuberculosis [31, 58]. Traditionally the plant species is also known for its potent antihelmintic, hepatoprotective, hypoglycemic, antifungal, antibacterial, antiaging and antifatigue properties. Herbal formulations have been mainly prepared based on different concentrations S. *chirayita* extract to develop hypoglycemic, antifungal, antibacterial properties and such formulations are already implemented for the development of some ayurvedic medicines like Ayush-64, Mensturyl syrup, Diabecon and Melicon V ointment [12]. Research investigations clearly revealed that such plant formulations can act as active protectant of heart and can able to regulate the blood pressure effectively.

### Antidiabetic Property

**Fig. 2:**
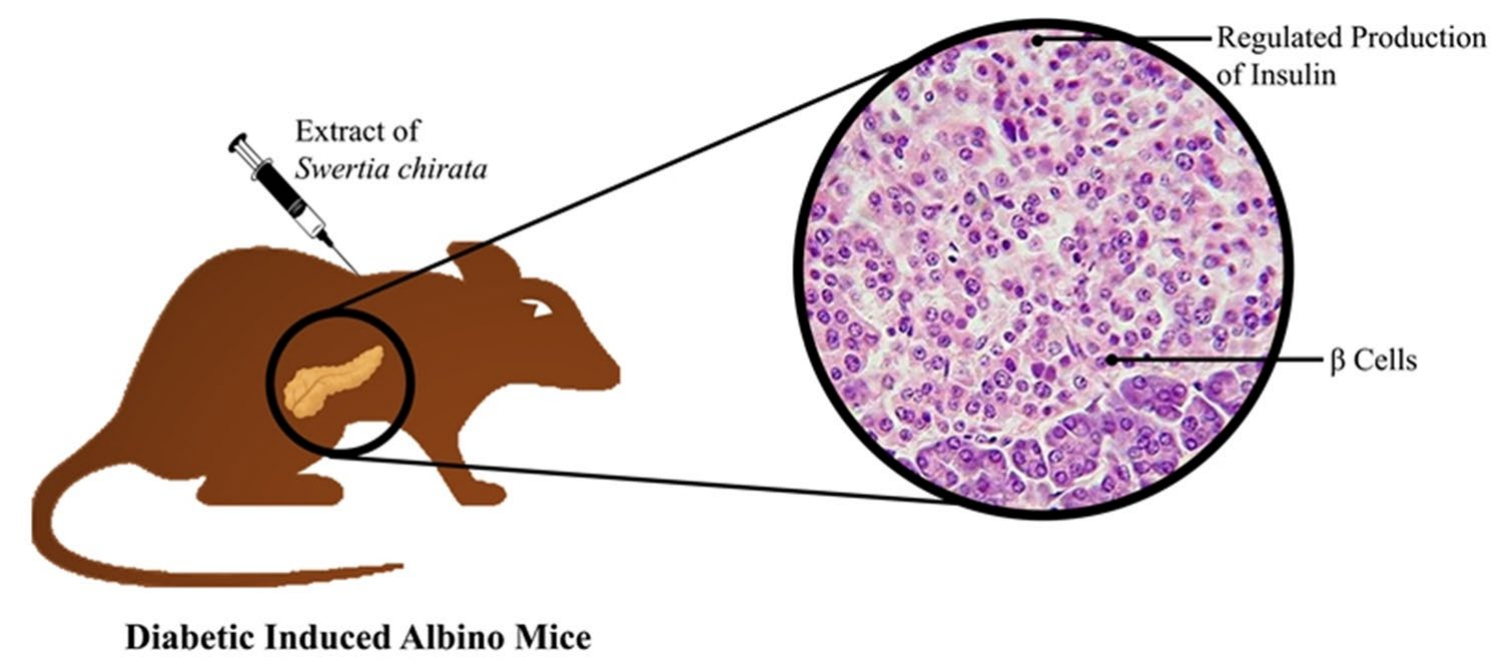
Schematic representation for regulated insulin production from pancreatic β-cells of *S*. *chirayita* extract injected mice

Different research investigations were already carried out to establish antidiabetic potential of the plant extracts, formulated from various parts of the herb. Presence of natural compounds like flavonoids, secoiridoids makes the plant extract highly effective to prevent hyperglycemic complications [54]. To investigate antidiabetic properties, insulin secretion from monolayers of BRIN-BD11 clonal pancreatic cells was investigated by Thomson et al. [26] in the influence of aquatic bark extract of *Swertia chirayita*. Stimulated concentration dependent insulin secretion and its enhanced action ware observed from such cell line in the presence of the extract. It was also attributed from their study that inhibition of protein glycation might help to prevent diabetic complications. Swerchirin, the most powerful xanthone isolated from the same plant extract having excellent blood sugar lowering activity and it was proved through experimental trials, being applied by different experimental models. The effects of structurally different hypoglycemic agents like tolbutamide, centpiperalone and swerchirin containing fraction (SW1), present in the same plant extract were identified using normal and diabetic rats, induced by streptozotocin [59, 60]. Compared to tolbutamide, SW1 exhibited improved blood glucose lowering effect in all rats except those having severe pancreatic damage. In another study, hexane extract of whole plant was evaluated for blood sugar lowering abilities by implementing CF male albino rats as experimental animal [61]. Swerchirin, one of the most important xanthone present in the plant extract was identified to make maximum influence in insulin release and maximum reduction of blood sugar level up to 41%. Non-alcoholic fatty liver disease is often responsible for formation of type 2 diabetes. S*. chirayita* plant extract was specifically analysed for glucuronidase inhibitory activities to establish its hepatoprotective potential [62]. Methanolic extract was prepared from the plant leafy shoots and it was identified with glucuronidase inhibition activity with IC50 value 162.84 ± 3.72 g/ml for swerchirin along with other influencer xanthone like mangiferin. Phoboo et al. [63] clearly identified the presence of mangiferin along with two other phytochemicals swertiamarin, amarogentin and their derivatives in crude plant extract. Among those components, mangiferin exhibited glucosidase and 2,2-diphenyl-1-picrylhydrazyl radical inhibition activity which mainly represents anti-hyperglycemic potential of the compound. Even oral administration of mangiferin with proper combination of standard hypoglycemic drugs, metformin and gliclazide was identified to cure symptoms of renal injury due to diabetic nephropathy (DN) [64]. During such experimental observation, type II diabetes induced male Sprague Dawley rats were treated with 28 days medication with such specific combination of drugs and significant decline in serum biochemical markers like glucose, urea and creatinine were clearly observed. In an experimental investigation, Kshirsagar et al. [65] extracted maximum amount of important secoiridoid glycoside swertiamarin and xanthone mangiferin from the plant species by adapting different extraction methods. Among various extraction techniques like static extraction, continuous shaking extraction and ultrasonic extraction, 24 h static extraction ensured maximum recovery of swertiamarin (256.98 mg/g) and mangiferin (155.76 ± 7.78 mg/g). Presence of most valuable bioactive compound gentianine, which is also known as active metabolite of swertiamarin was clearly identified in *Swertia chirayita* plant extract [66, 67]. Vaidya et al. [67] tried to investigate the role of gentianine behind anti-diabetic properties of swertiamarin. Significant improvement in adipogenesis linked expression of PPAR‐γ, GLUT‐4 and adiponectin upon gentianine treatment clearly revealed that the compound is responsible for anti‐diabetic activities of swertiamarin. Amarogentin, another important phytochemical present in all forms of the plant extract is also responsible for hypoglycemic activity. Amarogentin, which is mostly used in different forms of commercially available drugs was reported to be present 0.15% in S. *chirayita* species [68]. Knowing the potential of the compound, Keil et al. [69] developed root culture of the plant species in 2-L propeller stirred tank bioreactor system and a 15-fold improvement in amarogentin content was observed in the media when Tween 20 was implemented at 1.3% (v/v) during root permeabilisation treatment. How *S. chirayita* plant extract, containing such important phytochemicals is effective for regulated production of insulin from pancreatic β-cells of experimental model like mice is schematically represented in Fig. 2. In an experimental trial with albino Wistar rats, Kavitha and Dattatri [25], evaluated the antidiabetic activity of S. *chirayita* plant extract with comparison to standard oral drug glibenclamide. It was concluded that existence of mangiferin in plant stem part is mainly responsible for plants antidiabetic activity. Both crude aqueous extract and 12% ethanolic extracts of S. *chirayita* were clinically analysed to prove hypoglycemic potential of the plant species by performing in vitro biochemical tests [63]. The study clearly revealed the existence of mostly three phytochemicals which are mangiferin, swertiamarin, amarogentin as well as their derivatives in such plant crude extract. Wan et al. [70]evaluated hypoglycemic potential of another similar *Swertia* spp. plant extract i.e. *Swertia kouitchensis* in different conditions. Inhibitory action of the plant extract was first tested on α-amylase and α- glucosidase activities. Streptozotocin induced (60 mg/kg) diabetic mice were used to identify the acute effect of the extract and even the analysis was also performed to identify long term effect of the extract for 4 weeks. Inhibitory activities of α-amylase, α- glucosidase were well exhibited by the plant extract and similarly, anti-hyperglycemic activities, antioxidant capacity in improved level were also expressed by the plant extract. Zheng et al. [71] clearly informed that oxidative stress plays a crucial role in improvement of obesity based insulin resistance as well as second type of diabetes. They identified strong antioxidant activity of xanthones which was extracted from *Swertia mussotii*, another species of *Swertia*. In order to understand the hypoglycemic potential of ethanolic extract of S. *chirayita*, streptozotocin- nicotinamide influenced wistar albino mice (30–35 gm b/w) model was implemented by Renu et al. [72] and excellent serum sugar, cholesterol as well as triglyceride reducing activity was observed during implementation of such extract. In a similar type of research investigation by Sekar et al. [24], 95% ethanolic extract and four hexane fraction extractions of *Swertiu chirayita* were tested using albino rats for blood sugar level lowering ability. The hexane fraction showed important properties of reducing blood sugar in both fed and tolbutamide influenced models relative to the appropriate controls. In another case study, Chandrasekar et al. [73] demonstrated that hexane fractions of plant (250 mg/kg, orally administrated during 28 days) had an positive impact in lowering blood sugar level in albino rats and better insulin secretion from β-cells of pancreas. Alam et al. [23] investigated antidiabetic potential of the plant leaf ethanolic extract and different other forms of the plant extracts like petether, dichloromethane and methanoloc extracts on *Swiss albino* mice in fasting condition. Improved hypoglycemic properties through reduction of blood glucose levels about 32% and 47.2% were respectively achieved by applying ethanolic leaf extract and pet-ether fraction. Mild to moderate hypoglycemic effect of the plant extract was identified when reductions in blood glucose level were achieved 14.1% and 15.9% respectively by applying dichloromethane and methanol fractions after 3 h of drug administration. More description related to evaluation of anti-diabetic potential of S. *chirayita* plant extract is represented in Table 1 with different case studies.

**Table 1:** Anti-diabetic properties of *Swertia chirayita*

Therapeutic properties evaluated	Part of plant used	Solvent used for extraction	Test organism or model	Outcomes	References
Hypoglycemic and hypolipidemic properties	Whole plant	Directly dried and grinded material was used	Diabetes patients	Glucose level, triglycerides level, cholesterol level and level of LDL cholesterol decreased 14.5%, 10.5%, 8.6%, 14.4% respectively	[74]
Anti-hyperglycemic properties	Root extract	Ethanol	Swiss albino Wister rats	Decreases glucose, insulin levels and also improves lipid levels almost similar to the result of standard drug	[75]
Anti-hyperglycemic Properties	Stem-bark	Ethanol and water	nSTZ-T2DM rats	Substantial reduction in fasting glucose, cholesterol, triglycerides level and improvement in hepatic glycogen content	[76]
Antidiabetic Properties	Fresh tender leaves	Distilled water	Streptozotocin induced diabetic albino rats	Blood glucose levels in diabetic rats were reduced from 250.62 ± 2.35 mg/dl to 86.5 ± 2.85 mg/dl on 21st day	[77]
Hypoglycemic effect	Whole plant	Percolation with 95% ethanol and further concentrated with n-hexane	Tolbutamide pre-treated albino rat	Reduction of 40% blood sugar level	[61]
Lowering blood sugar	Whole plant	Hexane fraction	CF rats	Glucose consumption and glycogen development by muscle was improved and Blood glucose level was lowered by stimulating insulin release	[78]
Anti-diabetic properties	Different part of the plant like root, shoot and leaf	Crude aqueous and 12% ethanol	–	Existence of phytochemicals like mangiferin, swertiamarin, and amarogentin and their derivatives with anti-hyperglycemia potential was proved in crude plant extracts	[63]
Antidiabetic properties	Whole plant	Methanol	–	Analytical assays validated the presence of swertiamarin, mangiferin, amaroswerin, sweroside and amarogentin in plant extract	[79]

### Associated Pharmacological Property

Apart from diabetics, the plant extract is well known for its hepatoprotective, antioxidant, anti-inflammatory, antimalarial, antihelmintic, anticarcinogenic, antiviral, cytotoxic and antimicrobial properties. Different types of paracetamol and galactosamine models have been implemented to analyze the hepatoprotective properties of *S. chirayita* plant extract. Nagalekshmi et al. [17] analyzed how the plant extracts develop safety to defend acute hepatotoxicity, imposed by paracetamol dosages of 150 (mg/kg) on Swiss albino mice. Significant protection against paracetamol induced hepatotoxicity was developed with oral feeding of specific doses (100–200 mg/kg) of *S. chirayita* extract. Similarly Karan et al. [80] observed methanol extracted plant derivatives with dosages 100 mg/kg body weight of experimental animals was effective to develop 81% and 78% protection against paracetamol and galactosamine induced toxicities respectively. Balasundari et al. [81] identified that plant extract works specifically against paracetamol induced toxicity in initial single-layer cultures of rat hepatocytes. Zhou et al. [20] specifically identified twenty-six isolated compounds from *Swertia chirayita* ethanolic extract and every such compound were analyzed in vitro condition to develop protection against hepatitis B 17 virus using HepG 2.2.15 cell lines. Researchers also analyzed the hepatoprotective property developed through the treatment of such plant extract is also linked with its antioxidant property.

Chen et al. [82] examined antioxidant activity of *Swertia chirayita* plant extract against CCl_4_-induced toxicity mice by applying several standard in-vitro methods. Standard procedures were applied in their research to examine the properties of malondialdehyde (MDA) and antioxidant enzymes which includes superoxide dismutase (SOD), glutathione (GSH) and catalase (CAT). Powder generated by evaporation and lyophilization of ethanolic extract of *Swertia chirayita* was diagnosed with potent antioxidant ability. Balasundari et al. [81] proposed that lethal cell injury against CCl_4_ and elevated development of lipid peroxidation compounds due to deficiency of antioxidant enzyme activities can be prevented by xanthones, isolated from *Swertia chirayita* plant extract. In vivo experiments using different animal models were conducted in this study to confirm antiradical properties of xanthones rich extract specifically to prevent toxic effects of free radicals. Sharma et al. [83] evaluated antioxidant capacity of the plant extract by analyzing its free radical scavenging potential (DPPH assay). Butylated hydroxy toluene was used to analyze antioxidant capacity of acetonic and methanolic plant extract. The result of EC50 value of methanolic extract was evaluated as 27.70 μg/ml, which was comparable to the result of 17.75 μg/ml for butylated hydroxytoluene extract. It clearly indicates that methanolic extract of the whole plant possesses potent antioxidant property. Suryawanshi et al. [84] hypothesized that the presence of flavonoids and secoiridoids mainly determines the antioxidant property of methanolic plant extract.

Das et al. [85] evaluated analgesic as well as anti-inflammatory property of ethanolic plant root extract by pharmacological screening of rat paw edema model, induced by carrageenan. Writhing test based on acetic acid induction and radiant heat tail-flick methods were implemented to determine analgesic property. Extract was effective enough to lower the development of edema with doses level 400 mg/kg while resulting 57.81% inhibition of edema volume after 3 h. The experimental investigations clearly suggest that the plant is also having analgesic and anti-inflammatory properties. Banerjee et al. [57] used acute, sub-acute and chronic male albino rat experimental models to evaluate xanthone derivative (1,5-dihydroxy-3,8-dimethoxy xanthone) from *Swertia chirayita* extract against inflammation. They concluded that activity of the plant derivative is relatively comparable to the standard drug diclofenac in terms of anti-inflammatory activity. Alam et al. [86] investigated analgesic activity of ethanolic and methanolic extract of various plant parts like leaf, stem using acetic acid induced mice model. Findings from such investigation clearly suggest that ethanolic extract from leaf and stem part of the plant showed reasonable writhing inhibition (p < 0.001).

Several researchers [87, 88] also investigated the efficiencies of plant derivatives for antimalarial activity. Banerjee et al. [57] clearly indicated that xanthones present in the plant extract is mainly reasonable for such antimalarial activity. Bhat and Surolia [27], examined the organic and aqueous solvent extracts of three plants including *Swertia chirayita* for antimicrobial activity and also against malaria, caused by *Plasmodium falciparum* FCK 2. The plant extracts were evaluated in terms of its antimalarial activity on thin blood smears followed by using [35S]-methionine incorporation into parasite proteins and the activity levels in terms of inhibition was quantified as 50% (IC50). The result obtained in their study with [35S]-methionine incorporation clearly suggests that plant extracts having potent antimalarial activity. They also concluded that purer compounds isolated from plant extract having potential antimalarial activity compared to crude plant extracts. Similarly the plant extract was evaluated for its anti-carcinogenic and cytotoxic activities. Saha et al. [31] made their effort towards identifying compounds which can able to give protection against cytotoxic, genotoxic and metabolic activities of environmental toxicants in order to prevent the overall risk for cancer. For that, they have investigated anticarcinogenic activity of *Swertia chirayita* Buch on DMBA imposed mouse skin carcinogenic model. The extract of the plant was purified to enrich with 'Amarogentin' and tested for the specific activity. Effects like inhibited cell growth and induced apoptosis were apparent from both crude and purified form of the plant extracts. Parallelly, in order to identify the cytotoxic effects, Gopalkrishna et al. [89] clearly studied antiviral activity of the same plant extract specifically to develop protection against herpes simplex viruses. In diluted condition (1:64), the crude plant extract suppressed HSV-1, formation of plaque at higher level of 70% and the effect was confirmed by HSV antigen expression and indirect immunofluorescence (IFA) test.

Even the widespread application of *Swertia chirayita* plant extract was also explored with respect to antihelmintic activity and antimicrobial activity. Iqbal et al. [29] examined crude aqueous extract (25 mg/ml) and methanolic extracts of *Swertia chirayita* in different conditions for its anthelmintic activities. Plant extracts in crude aqueous and methanolic form were tested against mature live *Haemonchus contortus*, present in sheep to identify anthelmintic activity. Complete mobility of isolated worms was inhibited by methanolic plant extract with concentration at 25 mg/ml and the result was fully comparable with the result, obtained by the treatment of standard anthelmintic agent levamisole at concentration 0.55 mg/ml. Rehman et al. [30] screened antibacterial properties of same plant extracts in aqueous as well as ethanolic form against some pathogenic bacteria from clinical sources by applying Kirby Bauer’s disk diffusion method. The achieved results were evaluated against the application of standard antibiotic drug on the basis of zone of inhibition formation. Antibacterial effect of S. *Chirayita* was confirmed against some of the gram negative bacteria like *K. pneuomoniae, P. vulgaris* and *E. coli*. Similarly antibacterial activities of four plants including *Swertia chirayita* (stem) in the form of methanolic and aqueous extract were screened by Khalid et al. [90], specifically to inhibit gram positive bacteria like *Staphylococcus aureus* (ATCC 6538), *Entereococcus faecalis* (ATCC 14,506) and *Bacillus subtilis* (ATCC 6633). Positive inhibitory effect of the plant extract was achieved against *Bacillus subtilis* (ATCC 6633). Sultana et al. [91] evaluated the antibacterial potential of rectified spirit extracts, developed from fresh stem part of the plant by using disc diffusion method (concentration: 200 µg/disc) against few gram positive and gram negative bacteria. Observations were highly comparative with respect to standard antibiotic Kanamycin and significant antibacterial activity was observed against *Bacillus megaterium*, *Bacillus subtilis*, *Salmonella typhi-A*, *Shigella flexeneriae* and *Klebsiella* spp. Ahirwal et al. [92] evaluated both antibacterial and antifungal properties of same plant extract against standard drug Gentamycin and Amphotericin by using agar diffusion method. Presence of tannins and glycosides in plant extract was confirmed by phytochemical tests and presences of such phytochemicals were mainly responsible for the overall antimicrobial activity of the plant extract.

## Prospect and Limitations of Research

In Europe, USA and Japan, plant-based medicines got immense importance for the treatment of diseases for which no readymade medicine or treatment is directly available. As a result, in last one decade there has been a massive increase in utilization of such plant derived medicines in advanced western countries specifically like France and Germany [93, 94]. The inventions under modern herbal healthcare practices in developing countries like India are still under process. It is a common fact that the origins of maximum regularly used drugs under modern and traditional medicine are from plant sources. Almost 1% of the plant species from 250000 higher plants have been identified with medicinal properties and among them, some were specifically recognized for antidiabetic properties [5]. Such herbs have been identified by large potential of hypoglycemic activity but their effectiveness were not equally proved against all forms of diabetes and related complications. *Swertia Chirayita,* which is one among such ayurvedic plant, is well-known for its immense herbal and pharmaceutical applications. Value for the plant in national and global market has been constantly increasing. *S. Chirayita* from Indian origin and *S. japonica* and *S. pseudochinensis* from Japanise origin are considered under “elite” classes due to their huge therapeutic importance and revenue making possibilities [87]. Utilization of such plant in the form of crude drug has received growing demand in the Indian subcontinent (almost 400 tons/year) and such demand is predicted to increase 10% annually [87]. Still now the plant extract has been successfully implemented in the form of different compositions for remedial purposes of diabetics and other related disorders. Powdered form of the drug is normally utilized as a constituent of ‘sudarshan churna’, an ayurvedic tonic used in herbal medicinal method for management of all types of fevers [95]. So far no toxicity or serious side effects have been reported during various application of *Swertia Chirayita* plant extract although further extensive toxicological researches are necessary to establish total safety of the plant for human applications. Detail and time to time investigation of toxicological and mutagenic properties of different plant derivative compounds from *Swertia Chirayita* are also necessary as mutagenic and cytotoxic properties can be induced in such medicinal plants especially during long period utilization [96]. Despite of the long term recognition for various potent pharmacological activities, the pant does not possess enough scientific evidences regarding safety evaluation. In some cases, herbal formulations generated with active ingredients from such plant extract to defend specific diseases, were not well executed or established. Extensive investigation on antidiabetic mechanism, developed during application of diverse animal models is also absent for such plant species. In order to standardize the plant extract as medicinally valuable product and to analyze its therapeutic efficiency, proper identification of active components with their molecular interaction is highly necessary. Antidiabetic effectiveness and sensitivity evaluation of such plant extract against humans can be established by some extensive screening practices like oral glucose tolerance test (OGTT), fasting plasma glucose (FPG) test and random capillary blood glucose (RCBG) test. Even more specifically with RCBG test, Aspartate aminotransferase (AST) and Alanine aminotransferase (ALT) tests are also necessary to identify the condition of liver when the patient is under treatment with such plant extract. A series of histopathological studies of patient are needed to confirm the effect of plant extract on recovery of beta cells activity in terms of normal insulin production. Techniques like pre-fractionation of plant extracts can reduce the difficulties associated to the standardization of some screening methods [97].

The cultivation of herbal plants in hilly areas generates good business possibilities for local drug development organizations [98]. Under traditional medicinal practices, such type of herbal medicines were commonly implemented by general people in small quantities but in recent times large demand for such plant species is generated due to its extended commercialization and subsequent exploitation [99]. Now, extinction of such plant species by human interferences is estimated 100–1000 times faster than natural extinction and became a big threat for natural bio-resources [100]. Already wild population of many such plant species including *Swertia Chirayita* are reduced to a large extent due to developmental activities of Himalayan region. Due to multiple applications as traditional medicinal drug, the demand of the plant has been largely increased for both national and international trading and it causes rapid depletion of the plant wild populations [12]. Unlikely the wild medicinal plants have been extensively collected by untrained workers from the wild state. Such unskilled practices comes up with removal of whole plants, bulbs, rhizomes, tubers and roots before planting seeds and hence diminishes possibilities of regeneration of same plant species in the environment [99]. On the other side, over-exploitation practices of these species from the natural habitat caused substantial depletion of bio-resources and gradual loss of other associated species. Due to that fact, Indian National Medicinal Plant Board considered the plant among priority plant to support its conservation (https://www.nmpb.nic.in). Hence proper conservation practices of such critically endangered medical plant became essential to ensure its sustainability. The strategies like micropropagation, cryopreservation, utilization of suitable bioreactor systems which supports modern plant tissue culture techniques, commercial productions and conservation of such endangered herbs, can play effective role to improve such situation. In vitro efficient culture technique like micropropagation has received increased attention in recent years as most feasible method for mass proliferation of such endangered medicinal plants [101]. In order to endorse extended production and ex situ conservation of *S. chirayita*, Balaraju et al. [102] tried to upgrade the procedure for fast micropropagation by implanting explants of shoot tips from in vitro growing seedling. In recent times, in vitro propagation using somatic embryogenesis gained immense attention as most necessary technique for rapid development of uniform plants for investigation and also for its extended production [103]. The technique can be ideally used for formation of artificial seeds, as a complementary to the natural seeds [16]. For ideal preservation of critically endangered medicinal plant like *S. chirayita*, Kumar and Chandra, [16] developed most efficient plant regeneration protocol through induction of somatic embryo and formation of artificial seeds using in vivo leaf explants. Above all, spreading awareness about scientific protection, conservation and management of endangered medicinal plants among herbal plant collectors was already started from forest authority [99]. To ensure sustainable harvesting of such herbs, policies of rotational harvesting from demarcated areas have been also considered by concern agencies.

## Conclusion

Pharmacological importance of medicinal herb, *Swertia chirayita* is growing day by day due to its valuable phytochemical constituents. Its beneficial effects have largely been extended for management of diabetics and several other associated disorders. Although, the lack of sufficient evidence on safety assessment mainly hinders its large scale commercial applications on humans. Still large numbers of toxicological and histopathological studies are needed to be performed in order to understand its mechanism and standardize the plant product against human ailments. Conservation of such endangered plant species is another biggest concern as increasing national and international demand comes up with illegal overharvesting and unscrupulous collection of such wild plant species which finally leads to drastic depletion of the plant populations. Adaptation of some advanced plant biotechnological techniques like micro-propagation, hairy root technologies and synthetic seed production can help in surplus supply of such plant species to meet its future demand.
